# Effects of *N*-Glycosylation Site Removal in Archaellins on the Assembly and Function of Archaella in *Methanococcus maripaludis*

**DOI:** 10.1371/journal.pone.0116402

**Published:** 2015-02-20

**Authors:** Yan Ding, Kaoru Uchida, Shin-Ichi Aizawa, Kathleen Murphy, Alison Berezuk, Cezar M. Khursigara, James P. J. Chong, Ken F. Jarrell

**Affiliations:** 1 Department of Biomedical and Molecular Sciences, Queen’s University, Kingston, Ontario, Canada; 2 Department of Life Sciences, Prefectural University of Hiroshima, 562 Nanatsuka, Shobara, Hiroshima, Japan; 3 Department of Molecular and Cellular Biology, University of Guelph, Guelph, Ontario, Canada; 4 Department of Biology, University of York, Heslington, York, United Kingdom; Swiss Institute of Bioinformatics, SWITZERLAND

## Abstract

In *Methanococcus maripaludis* S2, the swimming organelle, the archaellum, is composed of three archaellins, FlaB1_S2_, FlaB2_S2_ and FlaB3_S2_. All three are modified with an *N*-linked tetrasaccharide at multiple sites. Disruption of the *N*-linked glycosylation pathway is known to cause defects in archaella assembly or function. Here, we explored the potential requirement of *N*-glycosylation of archaellins on archaellation by investigating the effects of eliminating the 4 *N*-glycosylation sites in the wildtype FlaB2_S2_ protein in all possible combinations either by Asn to Glu (N to Q) substitution or Asn to Asp (N to D) substitutions of the *N*-glycosylation sequon asparagine. The ability of these mutant derivatives to complement a non-archaellated *ΔflaB2_S2_* strain was examined by electron microscopy (for archaella assembly) and swarm plates (for analysis of swimming). Western blot results showed that all mutated FlaB2_S2_ proteins were expressed and of smaller apparent molecular mass compared to wildtype FlaB2_S2_, consistent with the loss of glycosylation sites. In the 8 single-site mutant complements, archaella were observed on the surface of Q2, D2 and D4 (numbers after N or Q refer to the 1^st^ to 4^th^ glycosylation site). Of the 6 double-site mutation complementations all were archaellated except D1,3. Of the 4 triple-site mutation complements, only D2,3,4 was archaellated. Elimination of all 4 *N*-glycosylation sites resulted in non-archaellated cells, indicating some minimum amount of archaellin glycosylation was necessary for their incorporation into stable archaella. All complementations that led to a return of archaella also resulted in motile cells with the exception of the D4 version. In addition, a series of FlaB2_S2_ scanning deletions each missing 10 amino acids was also generated and tested for their ability to complement the *ΔflaB2_S2_* strain. While most variants were expressed, none of them restored archaellation, although FlaB2_S2_ harbouring a smaller 3-amino acid deletion was able to partially restore archaellation.

## Introduction

*N*-glycosylation is a prevalent protein modification found in all three domains of life in which the attachment of the glycan is via the nitrogen atom in asparagine residues located in the acceptor glycoprotein [1–6]. General features of the *N*-glycosylation pathways are shared among the three domains [1–3,5,7,8]. The *N-*glycan precursor is first synthesized on a phosphorylated isoprene-based lipid carrier, either a dolichol derivative in Eukarya and Archaea, or an undecaprenol derivative in Bacteria, via the activities of glycosyltransferases. The assembled lipid-linked glycan is then flipped across a membrane, i.e. to face into the lumen of the endoplasmic reticulum in Eukarya or to the external face of the cytoplasmic membrane in Archaea and Bacteria. A signature enzyme of the pathway, the oligosaccharyltransferase (OST), transfers the completed glycan *en bloc* from its lipid carrier to select Asn residues in target proteins, although further sugars can still be added to the protein-bound glycan. The Asn residue to which the *N*-glycan is attached is usually located in an Asn-Xaa-Ser/Thr sequon (Xaa cannot be Pro), although in some Bacteria, i.e. *Campylobacter* spp., a negatively charged amino acid residue is also needed at the-2 position. Between the two prokaryotic domains, Bacteria and Archaea, *N*-glycosylation appears to be much more widespread among Archaea, where 166 of 168 sequenced genomes examined contained at least one copy of a gene encoding the archaeal OST AglB [9]. A variety of archaeal proteins, mainly S-layer proteins and the subunits of surface structures including both pilins and archaellins (formerly archaeal flagellins [10]), have been shown to be modified with *N*-glycans [11–18]. Recent work on archaeal *N*-glycosylation systems has combined both structural and genetic methods, typically using archaellins and S-layer proteins as reporter proteins. Since the first archaeal glycosylation (*agl*) genes were identified in 2006 [14,19], this combined approach has focused on a few key archaeal model organisms [20]: *Methanococcus maripaludis* S2, *Methanococcus voltae* PS, *Haloferax volcanii* H53 and *Sulfolobus acidocaldarius* MW001 [1,6,11,12,15,16,21]. Interestingly, the *N*-glycosylation pathway is not essential for any of the three studied euryarchaeotes (*M. maripaludis* S2, *M. voltae* PS and *Hfx. volcanii* H53), as mutants carrying a deletion or insertional inactivation of *aglB* were readily obtained [14,19,22]. However, in the crenarchaeote *S. acidocaldarius* MW001, repeated attempts to delete or interrupt *aglB* were unsuccessful and only the integration of a second copy of *aglB* into the genome allowed for the deletion of the original *aglB* [23]. The key reported effects that result from perturbation or complete abolition of the *N*-glycosylation pathway in Archaea are on S-layer stability, growth of cells at high salinities and on archaellation and motility [18,19,22,24–30].

The archaellum (formerly archaeal flagellum) is the major motility apparatus found in Archaea [10]. It is a rotating appendage unrelated to the bacterial flagellum but bearing instead similarities to the bacterial type IV pilus. These similarities include homologous ATPase and membrane platform proteins involved in assembly of the structure as well as similarities in their major structural proteins [31–35]. The structural proteins, called archaellins, are made as preproteins with class III (type IV pilin-like) signal peptides which are removed by a specific prepilin peptidase-like enzyme (termed FlaK in methanogens or PibD in Sulfolobales and halophiles) [36–39]. Signal peptide removal is critical for incorporation of archaellins into the filament [37, 39]. In addition, it appears that archaellins are commonly modified with *N*-linked glycans and interruption of the normal *N*-glycosylation pathway leads to defects in archaella assembly or function [1,40]. In species where *aglB* has been deleted or insertionally inactivated (*M. voltae* PS, *M. maripaludis* S2, *Hfx. volcanii* H53), the cells are unable to make archaella. If the pathway is interrupted at steps that lead to a truncated glycan, there is an impairment in motility, although archaella are still made unless the truncation is too great [18,19,22]. In *M. maripaludis* S2 strain, archaella contain 3 archaellins: the major archaellins FlaB1_S2_ and FlaB2_S2_ form the filament while the minor archaellin FlaB3_S2_ comprises the hook region [41]. The three archaellins share sequence similarities including a class III signal peptide cleaved by FlaK, conserved N-terminal and C-terminal regions, and a hypervariable region in the middle [41]. The hypervariable regions of FlaB1_S2_, FlaB2_S2_ and FlaB3_S2_ are decorated at multiple positions with a unique tetrasaccharide [11]. Cells are archaellated if they carry deletions in *agl* genes that result in a *N*-glycan of at least two sugars, but non-archaellated if the deleted *agl* gene results in a glycan of only a single sugar or prevents *N*-glycosylation totally (as in an *aglB* deletion) [22,42,43]. These results could mean that the archaellins must be glycosylated at some or all of its glycosylation sequons by at least a two sugar glycan for those archaellins to be assembled into a structure. However, an alternative explanation is that the necessity for the glycosylation lies at a different step in archaella assembly.

**Fig 1:**
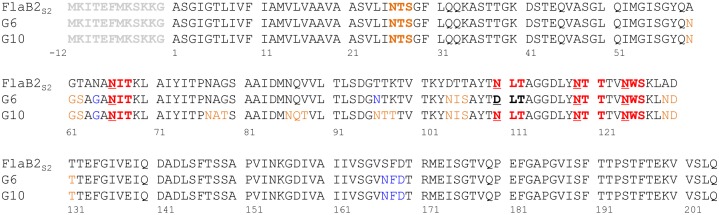
Protein sequence alignment of FlaB2_S2_, G6 (FlaB2_ΔRC_) and G10. Signal peptide is shown in grey; the first sequon ^26^NTS^28^ that is not occupied with *N-*glycan is shown in orange; the 4 occupied *N-*glycosylation sequons are shown in red; the 3-amino acid ^61^GTA^63^ deletion in 3AA is shown in green; extra sequons in the G6 and G10 are also shown in orange; sequences differences from FlaB2_S2_ in G6 and G10 that do not introduce new sequons are shown in blue.

A major goal of this study was to examine the requirement of *N*-glycosylation of the major archaellin FlaB2_S2_ for archaellation in *M. maripaludis* S2. FlaB2_S2_ has five *N*-glycosylation sequons, ^26^NTS^28^, ^66^NIT^68^, ^110^NLT^112^, ^119^NTT^121^ and ^124^NWS^126^. The first sequon ^26^NTS^28^ located in the N-terminal conserved region was previously reported to be unoccupied with glycan while the remaining four, located in the hypervariable region, were modified with tetrasaccharide (Fig. 1, [11]). For these experiments, we eliminated the 4 occupied sequons (^66^NIT^68^, ^110^NLT^112^, ^119^NTT^121^ and ^124^NWS^126^, designated as the 1^st^, 2^nd^, 3^rd^ and 4^th^ *N*-glycosylation site, respectively) in all possible combinations (creating single-, double-, triple- and quadruple-site mutations in FlaB2_S2_). We also generated a series of FlaB2_S2_ scanning deletions in an attempt to determine regions of the molecule that were essential for assembly of archaella.

## Materials and Methods

### Strains and growth conditions

*M. maripaludis* S2 *Δhpt* (Mm900) [44], *M. maripaludis* S2 *Δhpt ΔflaB2*_*S2*_ (*ΔflaB2*_*S2*_ in short hereafter) [41] and all complemented strains, as well as *M. maripaludis* ΔRC (formerly *Methanococcus deltae* ΔRC [45,46]) were routinely cultured anaerobically in 125 mL sealed serum bottles containing 10 mL Balch medium III under an atmosphere of CO_2_:H_2_ (20:80) at 37°C with shaking [47]. Cells carrying a complementation plasmid were cultured in the presence of 2.5 μg/mL puromycin for plasmid selection [48]. For swarming assays, cells were inoculated onto plates of Balch medium III containing 0.25% (w/v) agar in the presence of 2.5 μg/mL puromycin [22]. *Escherichia coli* TOP 10 cells (Invitrogen Inc.), used for molecular cloning steps, were cultured at 37°C in Luria Broth (LB) medium with shaking or on LB plates (containing 1.5% w/v agar) in the presence of 100 μg/mL ampicillin for plasmid selection. Strains and plasmids used in this study are listed in Table 1.

**Table 1:** Strains and plasmids used in this study.

Strains		References
***M. maripaludis***		
*M. maripaludis S2 Δhpt* (Mm900)		44
*M. maripaludis* S2 *Δhpt ΔflaB2_S2_*		41
*M. maripaludis ΔRC*		46
***E. coli***		
*E. coli* TOP10		Invitrogen Inc.
**Plasmids**		
pCR2.1-TOPO	TA cloning vector, Amp^r^, Kan^r^	Invitrogen Inc.
pKJ902	flaB2_S2_ in vector pCR2.1-TOPO	This study
pCR2.1-TOPO-*flaB2_S2_* derivatives	Mutant *flaB2_S2_* genes * in vector pCR2.1-TOPO	This study
pWLG40	*hmv* promoter-lacZ fusion plus Pur^r^ cassette; Amp^r^	48
pKJ1064	*flaB2_S2_* in shuttle vector pWLG40 under a *hmv* promoter	This study
pWLG40-*flaB2_S2_* derivatives*	Mutant *flaB2_S2_* genes * in vector pWLG40	This study

* Please refer to Table 2

### Construction of mutant *flaB2*_*S2*_ genes using site-directed mutagenesis (SDM)

To generate mutant *flaB2*_*S2*_ genes, the wildtype *flaB2*_*S2*_ gene was first cloned into the pCR2.1-TOPO-TA vector (Invitrogen Inc.) to create pKJ902. This pCR2.1-TOPO-*flaB2*_*S2*_ and its derivatives were used as template to generate the mutants listed in Table 2. The wildtype *flaB2*_*S2*_ gene used in cloning was generated by PCR using the complementation primers listed in Table 3 and genomic DNA from Mm900 as template.

**Table 2:** Mutated FlaB2S2 derivatives generated in this study.

Mutated FlaB2 derivatives	Description
**Control**	
WT	Wildtype *flaB2_S2_* from *M. maripaludis* S2
**Mutated FlaB2 derivatives containing mutations at *N*-glycosylation sites**	
**N to Q single-site mutation**	
Q1	FlaB2_S2_ N66Q
Q2	FlaB2_S2_ N110Q
Q3	FlaB2_S2_ N119Q
Q4	FlaB2_S2_ N124Q
**N to D single-site mutation**	
D1	FlaB2_S2_ N66D
D2	FlaB2_S2_ N110D
D3	FlaB2_S2_ N119D
D4	FlaB2_S2_ N124D
**N to D double-site mutation**	
D1,2	FlaB2_S2_ N66D N110D
D1,3	FlaB2_S2_ N66D N119D
D1,4	FlaB2_S2_ N66D N124D
D2,3	FlaB2_S2_ N110D N119D
D2,4	FlaB2_S2_ N110D N124D
D3,4	FlaB2_S2_ N119D N124D
**N to D triple-site mutation**	
D1,2,3	FlaB2_S2_ N66D N110D N119D
D1,2,4	FlaB2_S2_ N66D N110D N124D
D1,3,4	FlaB2_S2_ N66D N119D N124D
D2,3,4	FlaB2_S2_ N110D N119D N124D
**N to D quadruple-site mutation**	
D1,2,3,4	FlaB2_S2_ N66D N110D N119D N124D
**Mutated FlaB2_S2_ with extra *N*-glycosylation sequons**	
G6	FlaB2_S2_^60^AGT^62^ to ^60^NGS^62^, ^104^DDT^106^ to ^104^NIS^106^, N110D, A129N
G10	FlaB2_S2_^60^AGT^62^ to ^60^NGS^62^, G79T, V88T, ^96^TTK^98^ to ^96^NTT^98^, ^104^DDT^106^ to ^104^NIS^106^, A129N
**Ten-amino acid scanning deletions**	
Δ2–10	FlaB2_S2_ missing ^2^SGIGTLIVF^10^
Δ4–10	FlaB2_S2_ missing ^4^IGTLIVF^10^
Δ11–20	FlaB2_S2_ missing ^11^IAMVLVAAVA^20^
Δ21–30	FlaB2_S2_ missing ^21^ASVLINTSGF^30^
Δ31–40	FlaB2_S2_ missing ^31^LQQKASTTGK^40^
Δ41–50	FlaB2_S2_ missing ^41^DSTEQVASGL^50^
Δ51–60	FlaB2_S2_ missing ^51^QIMGISGYQA^60^
Δ61–70	FlaB2_S2_ missing ^61^GTANA**NIT**KL^70^ ^a^
Δ71–80	FlaB2_S2_ missing ^71^AIYITPNAGS^80^
Δ81–90	FlaB2_S2_ missing ^81^AAIDMNQVVL^90^
Δ91–100	FlaB2_S2_ missing ^91^TLSDGTTKTV^100^
Δ101–110	FlaB2_S2_ missing ^101^TKYDTTAYT**N** ^110^ ^a^
Δ111–120	FlaB2_S2_ missing ^111^ **LT**AGGDLY**NT** ^120^-^a^
Δ121–130	FlaB2_S2_ missing ^121^ **T**TV**NWS**KLAD^130^ ^a^
Δ131–140	FlaB2_S2_ missing ^131^TTEFGIVEIQ^140^
Δ141–150	FlaB2_S2_ missing ^141^DADLSFTSSA^150^
Δ151–160	FlaB2_S2_ missing ^151^PVINKGDIVA^160^
Δ161–170	FlaB2_S2_ missing ^161^IIVSGVSFDT^170^
Δ171–180	FlaB2_S2_ missing ^171^RMEISGTVQP^180^
Δ181–190	FlaB2_S2_ missing ^181^EFGAPGVISF^190^
Δ194–204	FlaB2_S2_ missing ^194^STFTEKV VSLQ^204^
**Three-amino acid deletion**	
Δ3AA	FlaB2_S2_ missing ^61^GTA^63^
**Substitution mutant**	
SUB	FlaB2_S2_ having ^91^TLSDGTTKTV^100^ substituted with IIVSGVSFDT

The mutant *flaB2*_*S2*_ genes were first generated in pCR2.1-TOPO and then cloned into the *M. maripaludis* expression vector pWLG40.

^a^ bold letters: *N*-glycosylation sequons with the asparagine underlined

**Table 3:** Primers used in this study^a^.

Complementation primers		Notes
comp-F	CTAGATGCATGAAAATAACAGAATTCATGAAAAGCAAAAAAGGTGCTTC	*Nsi*I
comp-R	ACGTTCTAGATTATTGTAATGAAACTACTTTTTCAGTGAATGTTGAAG	*Xba*I
**SDM primers**		
**Ten-amino acid deletion primers**		
2-F	***ATTCATGAAAAGCAAAAAAGGTGCT***ATTGCAATGGTATTAGTTGCTGCAG	
2-R	***AGCACCTTTTTTGCTTTTCATGAAT***TCTG	
4-F	***ATTCATGAAAAGCAAAAAAGGTGCTTCTGGA***ATTGCAATGGTATTAGTTGCTGCAG	
4-R	***TCCAGAAGCACCTTTTTTGCTTTTCATGAAT***TCTG	
11-F	***GGAATTGGTACCTTGATTGTTTTT***GCAAGCGTTTTAATTAACACAAGCG	
11-R	***AAAAACAATCAAGGTACCAATTCC***AG	
21-F	***CAATGGTATTAGTTGCTGCAG***TTGCATTACAACAAAAAGCTTCAACAACTGG	
21-R	TGCAA***CTGCAGCAACTAATACCATTG***C	
31-F	***CGTTTTAATTAACACAAGCG***GATTCGATAGTACCGAACAGGTTGCAAGC	
31-R	GAATC*CGC* ***TTGTGTTAATTAAAACG***CTTGC	
41-F	***CAACAAAAAGCTTCAACAACTGG***TAAACAAATTATGGGTATTAGCGGATACC	
41-R	TTTA***CCAGTTGTTGAAGCTTTTTGTTG***	
51-F	GTA***CCGAACAGGTTGCAAGC***GGTTTAGGTACTGCTAACGCAAACATTAC	
51-R	TAAACC***GCTTGCAACCTGTTCGG***	
61-F	***GGGTATTAGCGGATACC***AAGCAGCAATCTACATAACTCCTAACGCAGG	
61-R	TGCTT***GGTATCCGCTAATACCC***ATAATTTG	
71-F	***GCTAACGCAAACATTACAAAATTA***GCTGCAATAGACATGAATCAGGTTG	
71-R	***TAATTTTGTAATGTTTGCGTTAGC***	
81-F	***CATAACTCCTAACGCAGGAAGT***ACACTTTCAGACGGAACTACAAAAACTG	
81-R	***ACTTCCTGCGTTAGGAGTTATG***TAG	
91-F	***GCAATAGACATGAATCAGGTTGTTTTA***ACTAAATACGATACTACCGCATACAC	
91-R	***TAAAACAACCTGATTCATGTCTATTGC***	
101-F	***CTTTCAGACGGAACTACAAAAACTGTT***CTAACTGCAGGTGGAGACCTTTAC	
101-R	***AACAGTTTTTGTAGTTCCGTCTGAAAG***	
111-F	***ACGATACTACCGCATACACAAAC***ACAACTGTAAACTGGTCAAAATTAGC	
111-R	***GTTTGTGTATGCGGTAGTATCGT***ATTTAG	
121-F	***GCAGGTGGAGACCTTTACAACACT***ACTACAGAATTTGGAATAGTTGAAATTC	
121-R	***AGTGTTGTAAAGGTCTCCACCTGC***AG	
131-F	***GTAAACTGGTCAAAATTAGCAGAT***GATGCAGATCTTTCATTTACAAG	
131-R	***ATCTGCTAATTTTGACCAGTTTAC***AG	
141-F	***GAATTTGGAATAGTTGAAATTCAA***CCAGTTATCAACAAAGGTGACATAG	
141-R	***TTGAATTTCAACTATTCCAAATTC***TG	
151-F	***GATCTTTCATTTACAAGTTCAGCA***ATTATTGTAAGCGGAGTTTCATTCG	
151-R	***TGCTGAACTTGTAAATGAAAGATC***TGC	
161-F	***CAACAAAGGTGACATAGTTGCA***AGAATGGAAATTTCAGGTACTGTTCAG	
161-R	***TGCAACTATGTCACCTTTGTTG***ATAACTGG	
171-F	***GTAAGCGGAGTTTCATTCGATACA***GAATTTGGTGCTCCAGGAGTTATTTC	
171-R	***TGTATCGAATGAAACTCCGCTTAC***	
181-F	***GGAAATTTCAGGTACTGTTCAGCCA***ACCACACCTTCAACATTCACTG	
181-R	***TGGCTGAACAGTACCTGAAATTTCC***	
194-F	***CATTCACCACACCT*** *T* *A* *A* ***ACATTCACTGAAAAAGTA***	
194-R	***TACTTTTTCAGTGAATGT*** *T* *T* *A* ***AGGTGTGGTGAATG***	
**Three-amino acid deletion primers**		
3aa-F	***ATGGGTATTAGCGGATACCAAGC***AAACGCAAACATTACAAAATTAGC	
3aa-R	***TGCTTGGTATCCGCTAATACCCAT***AATTTG	
**Ten-amino acid substitution (sub) primers**		
sub-F	***TGTAAGCGGAGTTTCATTCG***ATACAACTAAATACGATACTACCGC	
sub-R	***CGAATGAAACTCCGCTTACA***ATAATTAAAACAACCTGATTCATGTC	
***N*-glycosylation site mutation primers**		
Q1-F	***GTACTGCTAACGCA*** *C* *A* *A* ***ATTACAAAATTAGC***	
Q1-R	***GCTAATTTTGTAAT*** *T* *T* *G* ***TGCGTTAGCAGTAC***	
Q2-F	***CTACCGCATACACA*** *C* *A* *A* ***CTAACTGCAGGTGGAG***	
Q2-R	***CTCCACCTGCAGTTAG*** *T* *T* *G* ***TGTGTATGCGGTAG***	
Q3-F	***GTGGAGACCTTTAC*** *C* *A* *A* ***ACTACAACTGTAAACTG***	
Q3-R	***CAGTTTACAGTTGTAGT*** *T* *T* *G* ***GTAAAGGTCTCCAC***	
Q4-F	***AACACTACAACTGTA*** *C* *A* *A* ***TGGTCAAAATTAGC***	
Q4-R	***GCTAATTTTGACCA*** *T* *T* *G* ***TACAGTTGTAGTGTT***	
D1-F	***GTACTGCTAACGCA*** *G* *AC* ***ATTACAAAATTAGC***	
D1-R	***GCTAATTTTGTAAT*** *GT* *C* ***TGCGTTAGCAGTAC***	
D2-F	***CTACCGCATACACA*** *G* *AC* ***CTAACTGCAGGTGGAG***	
D2-R	***CTCCACCTGCAGTTAG*** *GT* *C* ***TGTGTATGCGGTAG***	
D3-F	***GTGGAGACCTTTAC*** *G* *AC* ***ACTACAACTGTAAACTG***	
D3-R	***CAGTTTACAGTTGTAGT*** *GT* *C* ***GTAAAGGTCTCCAC***	
D4-F	***AACACTACAACTGTA*** *G* *AC* ***TGGTCAAAATTAGC***	
D4-R	***GCTAATTTTGACCA*** *GT* *C* ***TACAGTTGTAGTGTT***	

^a^Underlined: restriction enzyme sites

Italic bold: reverse complementary sequences in primer pairs

Italics: mutated amino acid codon

Underlined in italics: mutated DNA base

To generate the mutant *flaB2*_*S2*_ genes that would encode proteins in which *N*-glycosylation sites were eliminated, the SDM protocol was employed [49]. Forward and reverse mutagenic primer pairs were designed with nucleotide changes located in the middle of the primer that would result in a change of the *N*-glycosylation sequon Asn residue (Table 3). Purified PCR products were digested with *Dpn*I to remove the template plasmid, repurified and then transformed into *E. coli* TOP10 competent cells. Plasmids extracted from the transformants were sequenced to confirm the mutation. Using this method, 8 single-site mutant *flaB2*_*S2*_ genes were generated that resulted in 4 N to Q single-site mutations and 4 N to D single-site mutations in their protein products. Double-site mutant *flaB2*_*S2*_ genes were then generated using the plasmids with the single-site changes in *flaB2*_*S2*_ as template. The same strategy was used to create the triple and quadruple glycosylation site mutant *flaB2*_*S2*_ genes. The multi-site mutant proteins all contained N to D changes only.

The *G6* (*flaB2*_*ΔRC*_) gene was amplified by PCR using *M. maripaludis* ΔRC whole cells as template with the complementation primers in Table 3 and subsequently cloned into pCR2.1-TOPO. The *G10* gene whose protein product contains additional glycosylation sites was chemically synthesized by GenScript USA Inc. (Piscataway, NJ).

For construction of the mutant versions of *flaB2*_*S2*_ whose protein products would contain scanning deletions, inverse PCR and overlapping primers was employed, again with pKJ902 as template. The forward primer was designed to contain the *flaB2*_*S2*_ gene sequence flanking the desired in-frame 30 bp deletion. The reverse primer was complementary to the forward primer sequence upstream of the deletion (Table 2). After *Dpn*I digestion, purified PCR products were transformed into *E. coli* TOP10 competent cells. Recombinant plasmids were extracted from the transformants and used as template for amplifying the *flaB2*_*S2*_ mutant genes by PCR with the complementation primers listed in Table 2. The smaller *flaB2*_*S2*_ mutant genes were identified by agarose gel electrophoresis of the PCR products which were also sequenced to confirm the deletion. The same protocol was used to generate the 3 amino acid deletion, ^61^GTA^63^, where the deletion of the 9bp resulted in the removal of an *Rsa*I restriction site. This allowed for the screening of the *flaB2*_*S2*_ gene in plasmids carried by transformants for the small deletion by digestion of subsequent *flaB2*_*S2*_ PCR products with *Rsa*I. Lastly, a mutant *flaB2*_*S2*_ gene encoding a mutated FlaB2_S2_ protein with a 10-amino acid deletion at ^91^TLSDGTTKTV^100^ had those amino acids replaced with a copy of the 10 amino acids at ^161^IIVSGVSFDT^170^, thereby generating a substitution mutant version of FlaB2_S2_ (SUB) that was still the same length as the wildtype version. All the mutant versions of *flaB2*_*S2*_ generated were sequenced to confirm the mutations.

### Construction of complementation vectors

To generate complementation plasmids for *M. maripaludis* S2, mutant *flaB2*_*S2*_ genes in the pCR2.1-TOPO vector were PCR amplified using complementation primers with an *Nsi*I restriction site incorporated into the forward primer and an *Xba*I site into the reverse primer (Table 2). After *Nsi*I and *Xba*I digestion, the PCR product was cloned into the shuttle vector pWLG40 where transcription of the cloned gene is under the control of the strong constitutive *hmv* promoter [48]. Mutant *flaB2*_*S2*_ genes in pWLG40 were sequenced to confirm the insert gene sequence. As a further control, plasmids were re-isolated from the complemented cells and re-sequenced.

### Complementation of a *ΔflaB2*_*S2*_ mutant using mutant *flaB2*_*S2*_ derivatives

To determine if the mutant FlaB2_S2_ proteins generated above could restore archaellation and motility in the *ΔflaB2*_*S2*_ mutant, recombinant pWLG40 plasmids carrying the various mutant *flaB2*_*S2*_ derivatives were transformed individually into the *ΔflaB2*_*S2*_ mutant using a PEG-based method [41,50]. Transformants were cultured in Balch medium III containing 0.25 μg/mL puromycin for plasmid selection [48].

### Western blot analysis of the *ΔflaB2*_*S2*_ mutant complemented with mutant *flaB2*_*S2*_ derivatives

Whole cell lysates of complemented cells carrying the various mutant *flaB2*_*S2*_ genes were separated by SDS-PAGE (15% gels) and then transferred onto an Immobilon-P membrane (Millipore Inc.) [51]. Mutant FlaB2_S2_ proteins were detected using chicken anti-FlaB2_S2_ specific primary antibody [41]. Horseradish peroxidase-conjugated rabbit anti-chicken immunoglobulin Y (Jackson Immuno Research Laboratories) was used as secondary antibody, and the blots were developed using Immobilon Western Chemiluminescent HRP Substrate (Millipore Inc.).

### Swarming assay of the *ΔflaB2*_*S2*_ mutant complemented with mutant *flaB2*_*S2*_ derivatives

Complemented *ΔflaB2*_*S2*_ strains carrying plasmids with mutant *flaB2*_*S2*_ genes encoding proteins having mutations at the various *N*-glycosylation sites were examined for motility using semi-solid swarm plates [22]. Briefly, the OD_600_ of an overnight cell culture was measured and adjusted to 1.0. Five microliters of the adjusted cell culture were inoculated onto semi-solid Balch medium containing 0.25% (w/v) agar using a micropipette in an anaerobic chamber by stabbing the tip into the agar. Plates were incubated in an anaerobic canister at 37°C for 4 or 6 days.

### Electron microscopy of the *ΔflaB2*_*S2*_ mutant complemented with mutant *flaB2*_*S2*_ derivatives

Complemented *M. maripaludis ΔflaB2*_*S2*_ cells carrying mutant *flaB2*_*S2*_ genes were collected from an overnight culture by centrifugation at 20 000 g for 1 min, washed with 2% (w/v) NaCl and resuspended in phosphate-buffered saline. Resuspended cells were loaded onto carbon-Formvar-coated copper grids and stained with 2% phosphotungstic acid, pH 7.0. Grids were examined in a Hitachi 7000 electron microscope operating at an accelerating voltage of 75 kV.

## Results and Discussion

### Generation of mutant *flaB2*_*S2*_ derivatives

While deletions in genes that affect *N*-glycosylation are known to cause severe defects in archaellation and motility [18,22,28,29], it is not clear if the defects are related directly to the inability of non-glycosylated archaellins or archaellins glycosylated with truncated glycans to assemble into archaella, or whether the glycosylation defect affected other steps in the assembly of archaella. For example, it may be that another protein critical for assembly of archaella, but not an archaellin, must be glycosylated in order to function properly. In *Hfx. volcanii* H53, changing the sequence of the major archaellin *flgA* at any of the 3 examined sequons so that the encoded amino acid changed from Asn to Gln led to mutant forms of the protein that could not rescue the swimming defect of an *flgA* deletion strain, suggesting that each glycosylation site was necessary for archaellation [18]. However, this is not the case for *M. maripaludis* S2. Previous work in this methanogen showed that a strain that had a spontaneous mutation in *flaB2*_*S2*_ which led to the loss of the 2^nd^ *N*-glycosylation site of the archaellin that is normally decorated with the *N-*linked tetrasaccharide, was, nonetheless, still archaellated and motile [43]. To examine the possible role that each *N*-glycosylation site, either alone or in combination with other sites, might have on archaella formation and motility in *M. maripaludis* S2, various mutant *flaB2*_*S2*_ genes whose products were lacking single to quadruple *N*-glycosylation sites either by Asn to Gln (N to Q) substitution, or Asn to Asp (N to D) substitution of the *N*-glycosylation sequon asparagine were generated and cloned into the complementation vector pWLG40 (Table 2). For these mutant constructs we used D or Q followed by a number to indicate that the change was N to D or N to Q with the number representing the site changed, i.e. Q1 indicates mutant FlaB2_S2_ with an N to Q substitution at the 1^st^ *N*-glycosylation site.

In addition, two other mutant genes, designated G6 and G10 (Fig. 1), whose products contain extra *N*-glycosylation sequons were generated and cloned into pWLG40. The G6 sequence encodes the wildtype FlaB2_ΔRC_ protein (i.e. FlaB2_ΔRC_ from *M. maripaludis* ΔRC). FlaB2_ΔRC_ and FlaB2_S2_ share 95% identity, with the differences almost exclusively confined to several *N*-glycosylation sites (Fig. 1). Compared to FlaB2_S2_, FlaB2_ΔRC_ shares 3 sites, plus it has 3 additional sequons, ^60^NGS^62^, ^104^NIS^106^, ^129^NDT^131^, but it is missing the 2^nd^ *N*-glycosylation site ^110^NLT^112^ in FlaB2_S2_. The G10 protein has all the sites present in FlaB2_S2_ and FlaB2_ΔRC_, with 3 additional sites created in the hypervariable region at sites requiring only minimal amino acid changes to generate a total of 10 possible sites (Fig. 1). G6 and G10 both have the ^26^NIS^28^ sequon in the N-terminal conserved region that is not occupied with *N-*glycan in FlaB2_S2_. After transformation of these recombinant plasmid pWLG40-*flaB2*_*S2*_ mutants into a *ΔflaB2*_*S2*_ mutant, the complemented strains were examined for expression of the mutant FlaB2_S2_ proteins, archaella formation and cell motility.

### Western blot analysis of the Δ*flaB2*_*s2*_ strain complemented with *flaB2*_*S2*_ derivatives containing mutations at *N*-glycosylation sites

**Fig 2:**
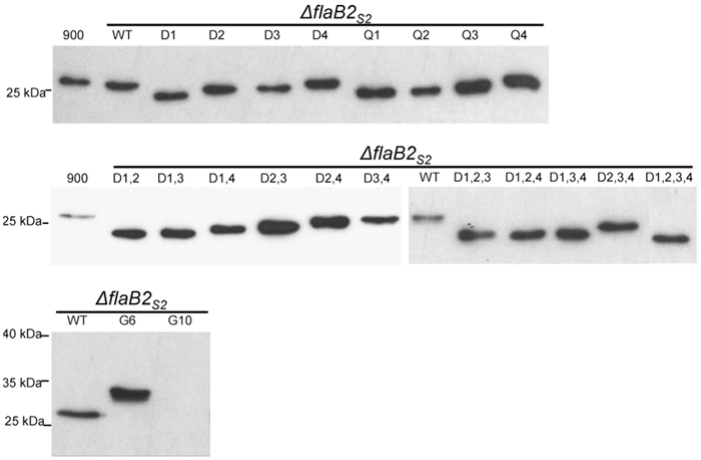
Western blot analysis of whole cell lysates of the *ΔflaB2*_*S2*_ strain complemented with *flaB2*_*S2*_ with mutations at various *N*-glycosylation sites. Mutant FlaB2_S2_ proteins missing single to quadruple *N*-glycosylation sites showed smaller apparent molecular mass than that of wildtype FlaB2_S2_. The G6 which has extra glycosylation sequons migrated slower than wildtype FlaB2_S2_. The expression of G10 was not detectable on this blot with normal exposure time. 900: wildtype *M. maripaludis* S2 *Δhpt*. WT: *ΔflaB2*_*S2*_ complemented with wildtype *flaB2*_*S2*_.

Western blots were run to detect the expression and stability of the various mutant versions of FlaB2_S2_ in the complemented *ΔflaB2*_*S2*_ mutant. As shown in Fig. 2, all mutant versions of FlaB2_S2_ except G10 were successfully expressed in the *ΔflaB2*_*S2*_ mutant. All mutant FlaB2_S2_ proteins were expressed in similar amounts and all appeared stable as judged by the general lack of any cross-reacting smaller molecular mass bands which could be indicative of protein degradation. The amount of the G10 version of FlaB2_S2_ detected in western blots was very low and could be only observed when blots were overexposed (data not shown). We have found previously that cells carrying mutations in any gene that prevents assembly of archaella (as in the *ΔflaB2*_*S2*_ mutant) often stop transcribing the *fla* operon after several sub-cultures in the laboratory. This then makes the complementation of the original gene deletion back to an archaellated state impossible [22]. For this reason, the presence of FlaE, whose gene is a downstream member of the *fla* operon, was also confirmed by western blot to ensure that the *fla* operon was still transcribed in the *ΔflaB2*_*S2*_ mutant during the course of the complementation experiments (data not shown) [41].

In general, mutant FlaB2_S2_ proteins missing *N*-glycosylation sites all had a smaller apparent molecular mass than that of wildtype FlaB2_S2_ when examined by western blotting, with the possible exceptions of D4 and Q4 which ran at very close to wildtype size. The greater the number of *N*-glycosylation sites eliminated in a particular FlaB2_S2_ mutant, the faster the mutant proteins migrated, i.e., single-site mutants had the largest apparent molecular mass, and the quadruple-site mutant D1,2,3,4 had the smallest. However, the 8 single-site mutants did not migrate as proteins of the same apparent molecular mass. Of the 8 mutants, D1 and Q1, both of which had the 1^st^ *N*-glycosylation site eliminated, had the smallest apparent molecular mass, while D4 and Q4 had the largest. Similar results were observed from the double-site and triple-site mutations. In the 6 double-site mutations, FlaB2_S2_ with D1,2 and D1,3 sites eliminated had the smallest apparent molecular mass, followed by FlaB2_S2_ with D1,4 and D2,3 sites eliminated, while the archaellin having the D2,4 and D3,4 sites eliminated migrated with the largest apparent molecular mass. In the triple-site mutants, FlaB2_S2_ with any of the 1^st^ *N*-glycosylation site eliminated (D1,2,3, D1,2,4 and D1,3,4) migrated at the same apparent molecular mass while FlaB2_S2_ with the other triple combination of sites eliminated (D2,3,4) migrated more slowly.

One possible explanation for the observed different electrophoretic mobilities is that mutant FlaB2_S2_ proteins lacking the same number of *N*-glycosylation sites have the same number of *N*-glycans attached but the attachment of *N*-glycan on some sequons might have effects on the local protein structure so that the glycoprotein is not able to be totally denatured by SDS, thus resulting in an unusual migration pattern. This unusual electrophoretic mobility has been observed in other similar studies and been the suggested explanation. Human erythropoietin (Epo) has 3 *N*-glycosylation sites, and the 3 single-site mutants generated by N to Q SDM showed uneven migrations on western blot, although all of the 3 mutants had the same theoretical molecular mass but differed only in the position of the *N*-glycans [52]. Similar uneven migration was also observed in the 4 single-site mutations of hepatitis C virus envelope protein E1 each missing one *N*-glycosylation site [53].

While local folding effects might explain the altered electrophoretic mobility, another possible explanation for this unusual western blot result is that elimination of the 1^st^ *N*-glycosylation site might interfere with the cell’s ability to *N*-glycosylate the remaining sites, resulting in FlaB2_S2_ where not all the remaining sequons are actually occupied (and so run as smaller molecular mass proteins). On the other hand, it may be that elimination of one or more glycosylation sites results in the attachment of glycan to the sequon ^26^NTS^28^ that is not glycosylated under our usual growth conditions. This could explain why, for example, the FlaB2_S2_ mutants that are missing the 4^th^ *N*-glycosylation site (D4 and Q4) showed a larger apparent molecular mass than the other single mutants and close to wildtype size. If D4 and Q4, missing the 4^th^ glycosylation site, now had *N*-glycan attached at the normally unused sequon, these mutant proteins would have an identical mass as the wildtype. There is precedent for glycosylation at one sequon influencing what happens at distant sites. For example, it has been reported for rabies virus glycoprotein that *N*-glycosylation at one sequon can influence processing of the *N*-glycans at a different site on the protein [54]. In *M. voltae* PS, the archaella are composed of 4 archaellins FlaA_Mv_, FlaB1_Mv_, FlaB2_Mv_ and FlaB3_Mv_, which share high sequence similarity in the N-terminal conserved region with FlaB1_S2_ and FlaB2_S2_ [41,55]. Interestingly, the first 40 amino acids in the mature FlaB1_Mv_ and FlaB2_Mv_ including the ^26^NTS^28^ sequon, are identical to those of FlaB2_S2_, but in the case of the *M. voltae* PS archaellins, the ^26^NTS^28^ sequon was found to be occupied with *N*-linked glycan [12]. Clearly, this region of the archaellin can be glycosylated and possibly the ^26^NTS^28^ sequon in FlaB2_S2_ might be able to be *N*-glycosylated in *M. maripaludis* S2 under different conditions.

The G6 mutant FlaB2_S2_ protein (identical to the FlaB2_ΔRC_) with additional glycosylation sequons compared to wildtype FlaB2_S2_ had a larger apparent molecular mass (~32 kDa) than wildtype FlaB2_S2_ (~27 kDa) in western blots. Since G6 has 6 *N*-glycosylation sites (excluding the ^26^NTS^28^ sequon), its larger apparent molecular mass suggests that at least some, and possibly all, of the extra sequons are, in fact, occupied since the mass of the tetrasaccharide is only 1036 Da [11]. Although the expression level of G10 was extremely low, on over-exposed western blots, the apparent molecular mass (37kDa) of this “artificially designed” glycoprotein was even larger than G6, indicating that AglB can recognize and transfer glycan to at least some of the newly introduced sequons in the hypervariable region.

### Electron microscopy of the Δ*flaB2*_*s2*_ strain complemented with *flaB2*_*S2*_ derivatives containing mutations at *N*-glycosylation sites

**Fig 3:**
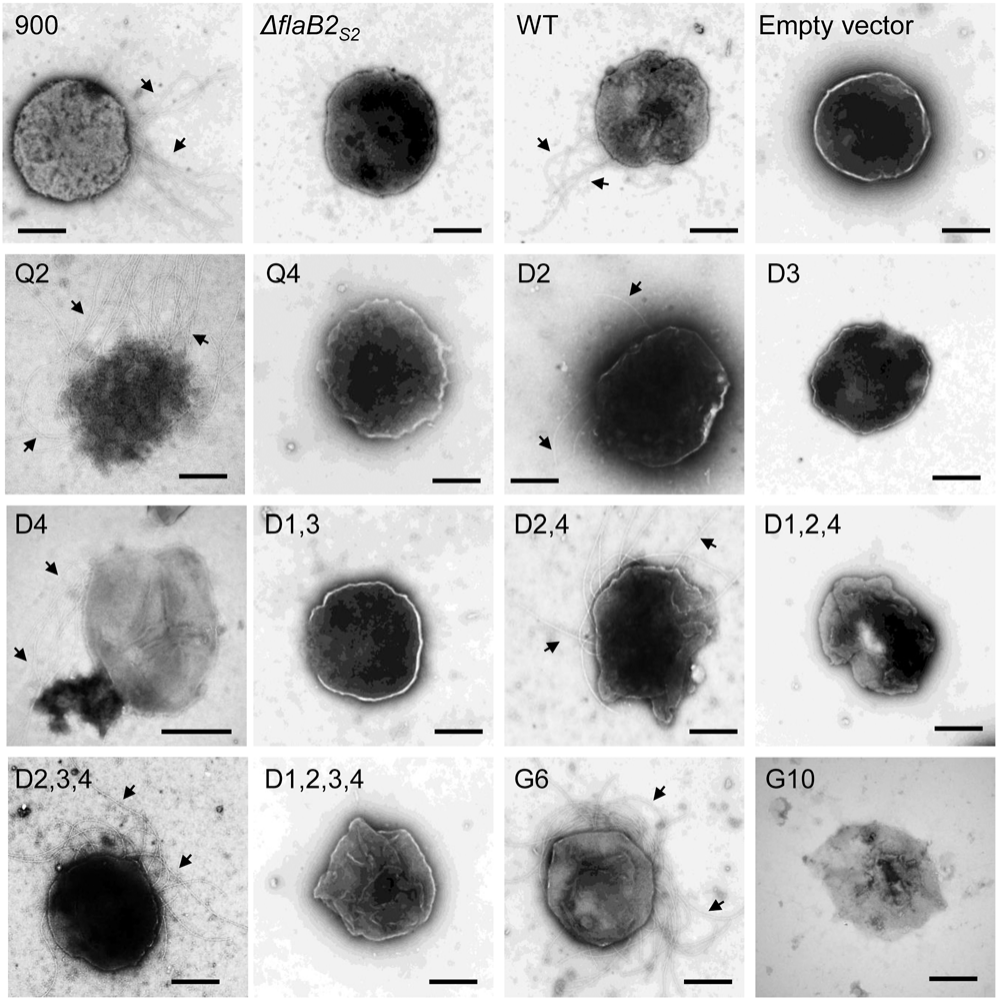
Transmission electron micrographs of select *ΔflaB2*_*S2*_ strain complemented with *flaB2*_*S2*_ with mutations at various *N*-glycosylation sites. Wildtype Mm900 cell (900) was archaellated, while the *ΔflaB2*_*S2*_ mutant was not. Wildtype FlaB2_S2_ protein expressed in *ΔflaB2*_*S2*_ restored archaellation (WT), but the empty vector could not. Archaella were observed on surface of Q2, D2, D4, D2,4, D2,3,4 and G6 complemented cells. Cells complemented with Q4, D3, D1,3, D1,2,4, D1,2,3,4 and G10 were non-archaellated. Archaella are indicated by arrows. Bar equals 500 nm.

Complemented cells carrying FlaB2_S2_ proteins having mutations at *N*-glycosylation sites were examined by transmission electron microscopy for archaellation, and the results are listed in Table 4. The majority of the complementations either restored archaellation to essentially all cells or were unable to restore archaellation to any cells. In only a couple of cases did the complementation lead to a population which contained roughly equal number of archaellated and non-archaellated cells (D1,2 and D3,4). Fig. 3 shows electron microscopy pictures of a number of selected complements (Q2, Q4, D2, D3, D4, D1,3, D2,4, D1,2,4, D2,3,4, D1,2,3,4, G6 and G10).

**Table 4:** Archaellation and swarming ability of complements bearing FlaB2_S2_ mutants at *N*-glycosylation sites.

Complements		Archaellation^a^	Motility
**Controls**	WT	++ (100%)	++
	Blank	- (0%)	-
**NQ single**	Q1	- (3%)^b^	-
	Q2	++ (100%)	++
	Q3	- (3%)^b^	-
	Q4	- (0%)	-
**ND single**	D1	- (3%)^b^	-
	D2	++ (100%)	++
	D3	- (0%)	-
	D4	++ (93%)	-
**ND double**	D1,2	+ (47%)	+
	D1,3	- (0%)	-
	D1,4	++ (97%)	++
	D2,3	++ (100%)	++
	D2,4	++ (100%)	+
	D3,4	+ (60%)	+
**ND triple**	D1,2,3	- (0%)	-
	D1,2,4	- (0%)	-
	D1,3,4	- (0%)	-
	D2,3,4	++ (100%)	++
**ND quadruple**	D1,2,3,4	- (0%)	-
**Additional sequons**	G6	++ (100%)	+++
	G10	- (0%)	-

^a^For each strain a minimum of 30 random cells were assessed for the presence or absence of archaella. Values in parentheses describe the percentage of cells with observable archaella.

^b^Only a rare cell was observed with archaella, typically very few in number and abnormally short.

The archaellation state of the 4 control strains was as expected. Wildtype Mm900 cells (900) were archaellated while the *ΔflaB2*_*S2*_ mutant strain was non-archaellated. Archaella were observed when the *ΔflaB2*_*S2*_ strain was complemented with the wildtype version of *flaB2*_*S2*_, but not when the *ΔflaB2*_*S2*_ strain was complemented with the empty vector pWLG40.

In the 8 single-site mutation complements, 3 mutant versions of FlaB2_S2_ (Q2, D2 and D4), could restore archaellation. In the 3 archaellated complements, Q2 and D2 had different amino acid substitutions at the same 2^nd^ *N*-glycosylation site. The N to D amino acid change at the 2^nd^ sequon generated in this study replicates the spontaneous mutation in *flaB2*_*S2*_ that we previously reported [43]. Both that spontaneous mutant and the complemented cells carrying the mutant *D2* gene generated in this study showed no impairment in archaellation or swarming motility [43]. These results indicate that missing the 2^nd^ *N*-glycosylation site alone does not significantly interfere with archaellation. In contrast, complementation of the *ΔflaB2*_*S2*_ strain with *flaB2*_*S2*_ lacking the 4^th^ *N*-glycosylation site differed markedly depending on what amino acid the original Asn was changed to; cells complemented with the D4 version had archaella under electron microscopy while cells complemented with the Q4 version did not (Fig. 3). None of the other single-site mutation complements (Q1, Q3, Q4, D1 and D3 (Fig. 3)) were considered archaellated, although in each of the Q1, Q3 and D1 complementations a rare cell with short archaella was observed (Table 4).

Among the 6 double-site mutation complements, all but the D1,3 version could assemble archaella (electron micrographs of D1,3 and D2,4 complemented cells are shown in Fig. 3). This was surprising since many of these double-site mutants contained eliminated sites which if deleted alone resulted in non-archaellated cells. In the 4 triple-site mutation complements, 3 complements D1,2,3, D1,2,4 and D1,3,4 were non-archaellated, while archaella could be assembled in D2,3,4 (Fig. 3). However, when all glycosylation sites were eliminated in the complementing version of *flaB2*_*S2*_ (D1,2,3,4), the *ΔflaB2*_*S2*_ cells were not able to assemble archaella. These results indicate that in *M. maripaludis* S2, archaella can be assembled using FlaB2_S2_ lacking as many as 3 out of the 4 glycosylation sites, as long as the first site remained intact (D2,3,4) but not when the archaellin is entirely non-glycosylated (D1,2,3,4).

In the two complementations where new sequons were introduced into FlaB2_S2_, different results were observed. In the G6 complemented cells, the *ΔflaB2*_*S2*_ strain were now archaellated, suggesting that the FlaB2_S2_ protein with extra *N*-glycan modifications in the hypervariable region could be incorporated into the archaellar filament by the archaella assembly apparatus in *M. maripaludis* S2. This was not unexpected since this version of FlaB2 already exists naturally in the archaellated *M. maripaludis* strain ΔRC. In contrast, no archaella were observed on the *ΔflaB2*_*S2*_ strain complemented with the G10 version. The G10 version had extra glycosylation sequons added to the internal hypervariable region of the protein. While this protein appeared to be modified at, at least, some of these additional sequons with glycan, judging from its higher apparent molecular mass in western blots, it was very poorly expressed in the cells under our normal growth conditions and this low expression may explain the lack of archaella observed by electron microscopy (Fig. 3).

### Swarming assays of complements with mutant *flaB2*_*S2*_ derivatives containing mutations at *N*-glycosylation sites

**Fig 4:**
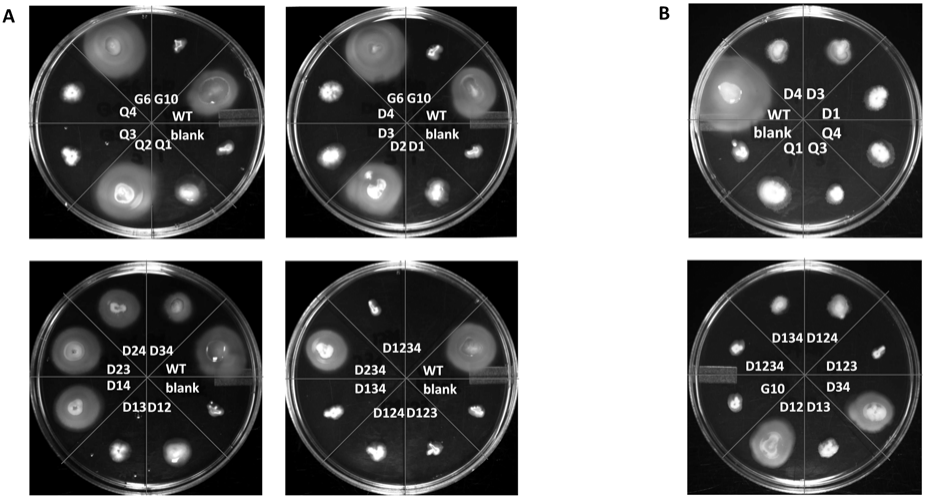
Swarming plates showing the motility of the *ΔflaB2*_*S2*_ strain complemented with *flaB2*_*S2*_ with mutations at various *N*-glycosylation sites. A. Plates were incubated at 37°C for 4 days. B. Complemented cells that did not show motility or showed poor motility after 4 days incubation were incubated a further 2 days.

In addition to the restoration of archaellation, the complemented cells were also examined for possible restoration of motility using semi-solid agar plates (Fig. 4A). Motility assay results are summarized in Table 4, which also incorporates the archaellation status of the complemented strains for comparison.

In general, complemented cells in which archaellation was restored were also motile on swarming plates and all of the complemented cells that were non-archaellated as determined by electron microscopy (D1, D3, Q1, Q3, D1,3, D1,2,3, D1,2,4, D1,3,4, D1,2,3,4 and G10) were also non-motile on swarm plates, even after an extra 2-day incubation (Fig. 4B). The unusual exception was the complementation with the D4 version of FlaB2_S2_ which was archaellated but non-motile (Fig. 3, Fig. 4A). However, among the motile complemented cells, the swarming diameter was not always returned to the wildtype level. Cells complemented with the *flaB2*_*S2*_ genes carrying D2, Q2, D1,4, D2,3 and D2,3,4, mutations swarmed out to a similar distance on semi-solid agar (swarming diameter of D2/ WT = 1.01±0.11, Q2/WT = 1.10±0.09, D1,4/WT = 0.93±0.14, D2,3/WT = 0.96±0.20, D2,3,4/WT = 1.08±0.22), while complements with the *flaB2*_*S2*_ genes carrying D1,2, D2,4 and D3,4 had a smaller swarming diameter (swarming diameter of D1,2/WT = 0.51±0.05, D2,4/WT = 0.68±0.13, D3,4/WT = 0.52±0.07). For the D1,2 and D3,4 complementations, the smaller swarming distance may be explained by the lower percentage of cells that were observed by electron microscopy to be archaellated, but in the case of the D2,4 all cells examined were archaellated. Interestingly, G6 appeared hyper-motile as it consistently swarmed further than cells complemented with the wildtype version of *flaB2*_*S2*_ (swarming diameter of G6/WT = 1.24±0.10).

In *Hfx. volcanii* H53, none of the 3 FlgA single-site mutation complements showed motility [18]. However, in this study, *M. maripaludis* S2 cells were still as motile as wildtype cells when the Δ*flaB2*_*S2*_ strains was complemented with *flaB2*_*S2*_ with the D2,3,4 changes, in which archaella were assembled using FlaB2_S2_ lacking 3 out of the 4 *N*-glycosylation sites. The structural protein (flagellin) of the functionally analogous bacterial swimming organelle, the flagellum, can also be modified with glycan, especially in Gram-negative bacteria, although the linkage is *O*-glycosidic rather than *N-*glycosidic [56,57]. The *O*-glycan modification in bacterial flagellin can be critical for flagella assembly, stabilization, motility, and even virulence in pathogens [56,58–60]. In *Pseudomonas syringae* pv. *tabaci*, flagellin FliC has 6 *O*-glycosylation sites, and single-site mutations in any of these sites resulted in various impairments in motility, while a mutant carrying mutations to eliminate all 6 *O*-glycosylation sites in FliC was non-motile [59]. The structural protein (pilin) from bacterial type-IV pili, structures which share several significant similarities with archaella [10,34], can also be *O*-glycosylated [61,62]. Elimination of *O*-glycosylation of type IV pilin resulted in reduced twitching motility in *Pseudomonas aeruginosa* 1244 and *P. syringae* pv. *tabaci* but did not interfere with pili assembly [62,63]. However, in *P. aeruginosa* 5196 in which a different *O*-glycan was attached to the type IV pilin PilA, *O*-glycosylation played critical roles in both type IV pili assembly and twitching motility [2,64].

The results obtained with the G6 complemented cells indicate that an increase in glycosylation can lead to hyper-motility. The western blots results indicate that the G6 version of FlaB2 is hyper-glycosylated compared to the wildtype FlaB2_S2_ version. Of all the complemented strains, only the G6 complement consistently demonstrated an increased zone of swarming compared to the wildtype. Interestingly, similar results were also observed in regards the *O*-glycosylation of flagellin in *Helicobacter pylori* [65] where *O*-glycosylation of the flagellins FlaA and FlaB with pseudaminic acid is essential for flagella assembly and cell motility [66,67]. A *H. pylori* mutant defective in deglycosylation of flagellins showed both hyper-*O*-glycosylation (3 fold more pseudominic acid) of FlaA as well as hyper-motility [65]. However, there is a limit to how many extra sequons can be added to archaellins since archaellin synthesis was very poor in the G10 complemented cells, even though the small amount of the G10 version detected was apparently modified at, at least, some of the extra sites.

The data obtained from the glycosylation site elimination mutants indicates that while no particular single site of glycosylation on FlaB2_S2_ is essential, nonetheless glycosylation at some site is necessary for archaella formation. In the case of the 4 triple-site mutants it is clear that glycosylation of only site 1 is sufficient for archaellation and motility. However, removal of the 1^st^ site did not always lead to non-archaellated cells as witnessed by the archaellated and motile cells observed in the D1,4 complementation, suggesting that glycosylation of FlaB2_S2_ at several different combinations of sites could be sufficient for incorporation of the subunits into functional archaella. In some ways, this is reminiscent of a situation in Wzc, a tyrosine autokinase essential for capsule formation in *E. coli*. Phosphorylation of tyrosine residues in the C-terminus of Wzc are necessary for its function but no single tyrosine is essential for phosphorylation and it was suggested that the overall level of phosphorylation rather than a precise combination of tyrosine residues accessible to phosphorylation is what is important for Wzc activity [68].

### Western blot analysis of the Δ*flaB2*_*s2*_ strain complemented with *flaB2*_*S2*_ scanning deletions

**Fig 5:**
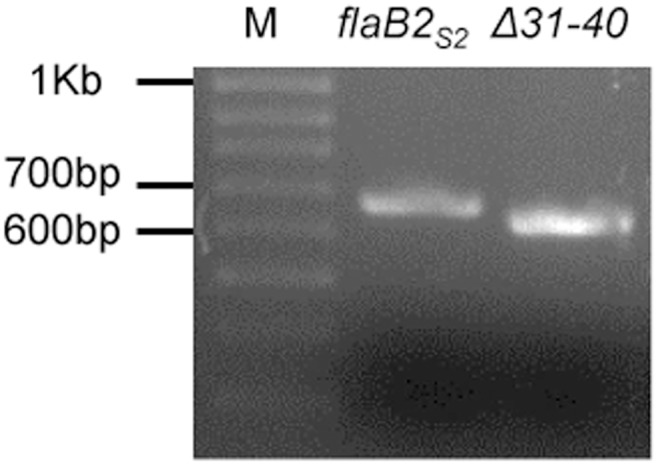
PCR screening of the *Δ31–40* scanning deletion. Following the deletion procedure, the *flaB2*_*S2*_ gene was amplified using *flaB2*_*S2*_ complementation primers and the PCR product analyzed by agarose gel electrophoresis along with the amplification product obtained with the wildtype *flaB2*_*S2*_ gene using the same primers. The scanning deletion is readily distinguished from wildtype *flaB2*_*S2*_ by the faster migration of its 30 bp smaller PCR product M: 100 bp DNA ladder; *flaB2*_*S2*_: PCR products using pKJ902 as template; Δ31–40: PCR products using plasmid isolated from one colony of the *Δ31–40* transformants as template.

To determine which regions of the FlaB2_S2_ protein are critical for archaella formation, a series of FlaB2_S2_ scanning deletion mutants that sequentially lacked 10 amino acids were generated in the complementation vector pWLG40 and transformed into the *ΔflaB2*_*S2*_ mutant. The scanning deletions in *flaB2*_*S2*_ were identified since they migrated slightly faster than the wildtype version of *flaB2*_*S2*_ in 0.8% agarose gels due to the 30 bp deletion (Fig. 5 shows an example for screening of *Δ31–40*). For the first 10 amino acids, two versions were generated. The first was deleted for amino acids 2–10 (named as Δ2–10), leaving the +1 amino acid which we thought might be important for successful cleavage of the 12 amino acid signal peptide. We also generated a 4–10 amino acid deletion (named as Δ4–10) since the +3 glycine of the mature protein is needed for signal peptide removal in archaellins of the related methanogen *M. voltae* PS [69].

**Fig 6:**
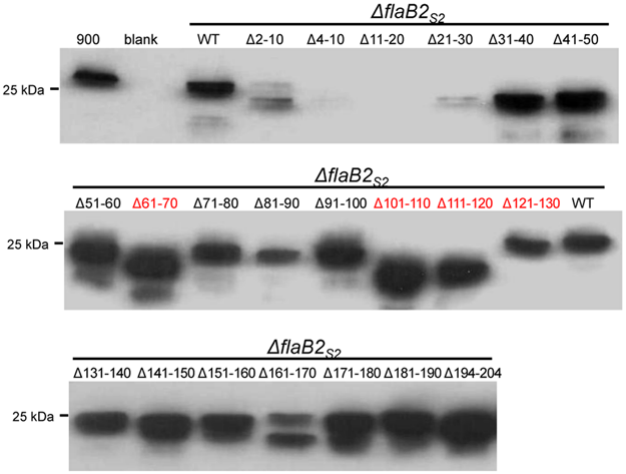
Western blot analysis of the *ΔflaB2*_*S2*_ strain complemented with *flaB2*_*S2*_ scanning deletions. Except for Δ4–10 and Δ11–20, all the FlaB2_S2_ scanning deletion proteins were expressed, although the expression level of Δ2–10 and Δ21–30 was relatively low. FlaB2_S2_ scanning deletion proteins Δ61–70, Δ101–110, Δ111–120 whose 10-amino acid deletion contains an *N*-glycosylation site (shown in red) had smaller apparent molecular masses due to the loss of *N*-glycan usually attached at this site. Δ121–130 missing the 4^th^ *N*-glycosylation site (shown in red) had unusual bigger apparent molecular mass than the other 3 mutants also missing *N*-glycosylation site.

Mutant FlaB2_S2_ proteins from whole cell lysates of the various complemented cells were detected using anti-FlaB2_S2_ specific antibody on western blot analysis, as shown in Fig. 6. In the 21 FlaB2_S2_ scanning deletions, 19 mutant proteins (all, except Δ4–10 and Δ11–20) were readily detected on western blot by anti-FlaB2_S2_ specific antibody, although the expression level of Δ2–10 and Δ21–31 was relatively lower compared to that of the other mutants. Evidence of some possible protein degradation was observed in the Δ2–10 and Δ161–170 FlaB2_S2_ as multiple lower molecular mass bands were detected in these two lanes. These results indicate that some of the mutant proteins were either not expressed or were unstable and degraded. Fourteen mutant proteins, including Δ21–30, Δ31–40, Δ41–50, Δ51–60, Δ71–80, Δ81–90, Δ91–100, Δ131–140, Δ141–150, Δ151–160, Δ161–170, Δ171–180, Δ181–190 and Δ194–204, had similar apparent molecular masses, which were smaller than FlaB2_S2_ from the wildtype strain Mm900 (900) or from the Δ*flaB2*_*S2*_ strain complemented with the wildtype version of *flaB2*_*S2*_. This is consistent with the fact that all these mutation proteins are 10 amino acids shorter than wildtype FlaB2_S2_. The upper band in the mutant Δ2–10 lane appears slightly larger than the neighboring WT lane. This might be due to the lack of processing of the archaellin signal peptide in this deletion since in *M. voltae* PS the +3 glycine position was essential for cleavage of the signal peptide by the pre-archaellin peptidase FlaK and this is missing in the Δ2–10 version of FlaB2_S2_ [69]. The presence of the signal peptide does not prevent the attachment of *N*-glycans [37] so this version of FlaB2_S2_ would be expected to have a full complement of attached *N*-glycans as well as the extra amino acids of the signal peptide contributing to make it run slightly larger in the western blots than the processed wildtype version of FlaB2_S2_.

The FlaB2_S2_ mutants Δ61–70, Δ101–110 and Δ111–120 had even smaller apparent molecular masses compared to the FlaB2_S2_ carrying other scanning deletions. This can be attributed to the fact that these three deletions result in the loss of one *N*-glycosylation sequon. Surprisingly, the scanning deletion that contains the remaining, 4^th^, glycosylation sequon, namely Δ121–131, does not follow this pattern. In this lone case, FlaB2_S2_ migration in western blots is slower than the other three deletion mutants missing *N*-glycosylation sites. This observation is consistent with the results of the *N*-glycosylation sequon mutation complementations. In the case of both single 4^th^ sequon mutants (D4 and Q4), FlaB2_S2_ had a larger apparent molecular mass than the other single-site mutations (Fig. 2), suggesting either that the lack of glycosylation at this site has unusual effects on the migration of FlaB2_S2_ in western blots or that when the 4^th^ glycosylation site is not available, the normally unoccupied ^26^NTS^28^ is now decorated with glycan, adding to the molecular mass. Further studies are necessary to confirm the glycosylation status of this ^26^NTS^28^ sequon in FlaB2_S2_ in these mutant proteins.

### Electron microscopy of the Δ*flaB2*_*s2*_ strain complemented with FlaB2_S2_ scanning deletions

To examine if Δ*flaB2*_*S2*_ cells could assemble archaella after being complemented with any of the FlaB2_S2_ scanning deletions, cells from each complementation were observed by transmission electron microscopy for the presence of archaella. All of the 21 10-amino acid scanning deletion complements were found to remain non-archaellated (data not shown) even though most of them produced FlaB2_S2_ detected by western blot. These results suggested that either all regions of the molecule were essential for archaella formation or that a certain critical archaellin length is important for the archaella filament to be assembled.

**Fig 7:**
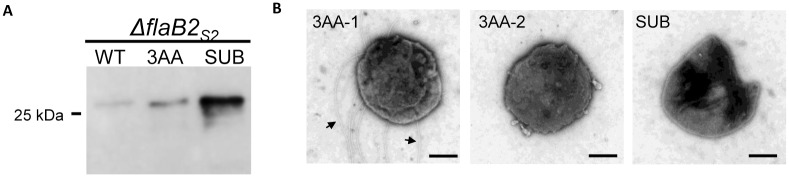
Analysis of the *ΔflaB2*_*S2*_ strain complemented with *flaB2*_*S2*_ carrying the 3-amino acid deletion (3AA) or the substitution version of *flaB2*_*S2*_ (SUB). A. Western blot analysis of the *ΔflaB2*_*S2*_ strain complemented with *flaB2*_*S2*_ carrying the 3-amino acid deletion (3AA) or the substitution version of *flaB2*_*S2*_ (SUB). 3AA and SUB had similar apparent molecular mass as wildtype FlaB2_S2_. B. Transmission electron micrographs of the *ΔflaB2*_*S2*_ strain complemented with the 3AA or SUB versions of FlaB2_S2._ In the case of the 3AA complemented cells, both archaellated cell (3AA-1) and non-archaellated cell (3AA-2) are shown. Arrows show the archaella. Bar equals 500 nm.

Since none of the FlaB2_S2_ scanning deletion mutants could restore archaellation, we suspected that the 10-amino acid deletion in the scanning deletions was too long for FlaB2_S2_ to be assembled into archaella. To address this, we created a shorter 3-amino acid deletion in the hypervariable region of FlaB2_S2_. This protein was detected by western blot at a similar apparent molecular mass as the wildtype FlaB2_S2_ (Fig. 7A). The examination of Δ*flaB2*_*S2*_ cells complemented with the Δ3AA version of *flaB2* by electron microscopy revealed that even with this short deletion, only approximately half of the complemented cells were archaellated (Fig. 7B). These results suggest that a 3-amino acid deletion might be the shortest deletion that FlaB2_S2_ could tolerate and still be assembled into archaella filaments.

We also tried to examine the possible length requirement of archaellins in a different way. In a FlaB2_S2_ that was already deleted for ^91^TLSDGTTKTV^100^, we inserted into this spot IIVSGVSFDT (originally from ^161^IIVSGVSFDT^170^), creating a FlaB2_S2_ hybrid that had amino acids 91–100 replaced with a second copy of amino acids 161–170 so that the resulting length of the FlaB2_S2_ (dubbed a substitution; SUB) was wildtype. Both the donor and the acceptor regions are located in the hypervariable region of FlaB2_S2_, and do not contain *N*-glycosylation sequons to minimally reduce the effects from disruption of the conserved regions that might be involved in subunit-subunit interaction or in glycosylation. The SUB protein was expressed in the complement cells and showed similar apparent molecular mass as that of WT protein, as expected (Fig. 7A). However, the SUB protein complement could not restore archaellation either (Fig. 7B), suggesting that the particular 10-amino acid sequence ^91^TLSDGTTKTV^100^ is critical for archaella assembly, despite the fact it is located in a hypervariable region of the molecule. We had anticipated that at least some of the scanning deletions covering the hypervariable region may have been tolerated and allow for formation of archaellation while those located in the conserved N-terminus believed to critical for subunit-subunit interactions in the filament would not be tolerated [70,71]. It is known that in the case of bacterial flagellins large internal deletions can be accepted; for instance in *E. coli*, the 493 amino acid flagellin can be reduced by internal deletions so that only the N-terminal 193 residues and the 117 C-terminal amino acids are required for filament formation [72]. In addition, sequences in the internal hypervariable region of bacterial flagellin can be replaced with completely unrelated sequences [73]. This is also true for archaellins in *Halobacterium salinarum* where both FLAG (8 amino acid peptide) and a gold-binding 12 amino acid peptide have been inserted into variable regions of different archaellins and these mutant proteins were still able to be assembled into archaella [74]. However, for type IV pilins, it has been shown in a number of studies that very small changes at key amino acids in the major pilins can result in instability of the pilins and ones that cannot assemble into pili [75–77].

Archaella are unique swimming organelles that are thought to be assembled like bacterial type IV pili but function like bacterial flagella by filament rotation [10,33,34,78]. So far little is known about details of the incorporation of individual archaellins into the archaella filament. *N*-glycosylation seems to be a common modification of archaellin, but the relationship between *N-*glycosylation and archaella assembly is unclear [1,40]. In this study, we investigated the effects of eliminating potential *N*-glycosylation sites as well as scanning deletions of the archaellin FlaB2_S2_ on archaella assembly and function in *M. maripaludis* S2. In *M. maripaludis* S2, functional archaella can be assembled using FlaB2_S2_ lacking as many as 3 out of 4 glycosylation sites (D2,3,4), but not when the archaellin is entirely non-glycosylated (D1,2,3,4). A hyper-*N*-glycosylated version of FlaB2_S2_ (G6) resulted in hyper-motile *M. maripaludis* S2 cells. Attempts to define essential and nonessential domains of the archaellin by scanning deletion analysis revealed that no contiguous 10 amino acid stretch could be deleted and still have the archaellin complement a Δ*flaB2*_*s2*_ strain back to an archaellated phenotype.
